# A failed attempt to conduct an individual patient data meta-analysis

**DOI:** 10.1186/2046-4053-3-97

**Published:** 2014-09-04

**Authors:** Gerald J Jaspers, Pieter LJ Degraeuwe

**Affiliations:** 1 Department of Pediatrics, Maastricht University Medical Centre, P. Debyelaan 25, PO Box 5800, 6202AZ Maastricht, The Netherlands

**Keywords:** Hypoxic-ischemic encephalopathy, Infant-newborn, Magnetic resonance spectroscopy, Meta-analysis as topic

## Abstract

A study-level meta-analysis has shown that proton magnetic resonance spectroscopy is a promising prognostic marker in neonatal hypoxic-ischemic encephalopathy. An individual patient data meta-analysis could yield a prognostic tool with improved accuracy enabling well-founded clinical decisions. Our request to share patient data remained unanswered by five out of 18 research groups. Another four declined collaboration for various reasons, including own reanalysis of the data, and lack of parental consent. With less than 40% of the individual patient data available, we refrained from pursuing the proposed study. As future patients may benefit from it, policies mandating data sharing should be introduced.

## Correspondence/Findings

End-of-life decisions and withdrawal of intensive care frequently precede death in newborns suffering from hypoxic-ischemic encephalopathy [1]. These decisions, taken in the neonatal intensive care unit, are to a large extent supported by neuroimaging and neurophysiological findings. According to a recent systematic review however, these prognostic tools, although promising, definitely lack precision [2]. In their meta-analysis, Thayyil and coworkers found that deep gray matter magnetic resonance biomarkers (especially measuring lactate/acetyl aspartate ratio) have good accuracy for predicting adverse outcome in neonatal encephalopathy [3]. Professor Wilkinson appropriately criticized this meta-analysis and suggested to perform an individual patient data meta-analysis [4], a challenge we accepted. The study protocol was published last year in Systematic Reviews [5].

As described in the study protocol, authors of all selected prognostic accuracy studies were contacted by email. They were offered the opportunity to comment on the study proposal. We acknowledged the challenge of the study but emphasized the known benefits of an individual patient data meta-analysis. We underlined the support we got from Prof. N. Robertson, an expert in the field. The ethical considerations were supported by our Medical Ethics Committee and we do have the opinion that the publication policy treated every contributory group fairly.

Five out of 18 approached research groups showed a favorable response and were ready to share raw patient data. To our surprise, five contacted groups did not answer our request for collaboration, even after two email reminders, in which we mentioned the positive reactions received until then. In four cases, our request was met with a refusal. In two cases, the main reason for noncooperation was lack of personnel or time to retrieve the data. We realized from the start that the effort asked for was substantial but worth making. In the future, mandatory release of all raw data of every published prognostic accuracy study could facilitate the process of individual patient data (IPD) meta-analysis. This is in line with the growing awareness that sharing data is an ethical obligation [6,7]. One author, contacted by phone, was reanalyzing his own data in collaboration with another research group (although they emailed: 'we do not currently have personnel available to look through all of our records’) and could therefore not share the raw data. This argument reminded us of a paper of Ioannidis wherein the author states that databases can be used as 'private goldmines not to be shared’ or as 'public commodity’ [8]. One author raised the issue of the lack of permission to go back to the original patient records. Although legislation might differ between countries, our medical ethics committee did not make an objection to the proposed IPD meta-analysis, under the express condition that the patient data are de-identified. It is hard to think that parents having consented for the original study would object to an IPD meta-analysis. We even imagine that parents would disagree with the waste of their children's data for secondary research.

All in all, even if we would be able to obtain all data from the nonresponders, 55% of the individual patient data at the very most will be available for the proposed IPD meta-analysis. So, we refrain from the proposed IPD meta-analysis and advocate instead a large prospective multicenter study to investigate the predictive value of MRS biomarkers simultaneously with other clinical, neuroimaging, and neurophysiological variables. This seems currently the best way to investigate whether a reliable prediction tool can be established for babies with neonatal encephalopathy.

Finally, while it is tempting to blame individual researchers for lacking a cooperative attitude, we have to acknowledge that there are currently no incentives for sharing data, and the debate of sharing clinical trial data is still ongoing. It is our hope that the general attitude about raw data availability will change and that funders and journals will make sharing practices mandatory for diagnostic and prognostic studies as well [9].

## Competing interests

The authors declared that they have no competing interests.

## Authors’ contributions

By mutual agreement GJJ and PLJD conceived the content of this letter. Both authors read and approved the final manuscript.
